# Identification of Key Genes and Prognostic Analysis between Chromophobe Renal Cell Carcinoma and Renal Oncocytoma by Bioinformatic Analysis

**DOI:** 10.1155/2020/4030915

**Published:** 2020-01-09

**Authors:** Hongwei Wu, Lijing Fan, Haiping Liu, Baozhang Guan, Bo Hu, Fanna Liu, Berthold Hocher, Lianghong Yin

**Affiliations:** ^1^Department of Nephrology, The First Affiliated Hospital of Jinan University, Jinan University, Guangzhou 510632, China; ^2^Department of Nephrology, The Second People's Hospital of Lianping County, Heyuan, China; ^3^Department of Medicine Nephrology, Medical Faculty Mannheim Heidelberg University, 68167 Mannheim, Germany

## Abstract

The present techniques of clinical and histopathological diagnosis hardly distinguish chromophobe renal cell carcinoma (ChRCC) from renal oncocytoma (RO). To identify differentially expressed genes (DEGs) as effective biomarkers for diagnosis and prognosis of ChRCC and RO, three mRNA microarray datasets (GSE12090, GSE19982, and GSE8271) were downloaded from the GEO database. Functional enrichment analysis of DEGs was performed by DAVID. STRING and Cytoscape were applied to construct the protein-protein interaction (PPI) network and key modules of DEGs. Visualized plots were conducted by the R language. We downloaded clinical data from the TCGA database and the influence of key genes on the overall survival of ChRCC was performed by Kaplan–Meier and Cox analyses. Gene set enrichment analysis (GSEA) was utilized in exploring the function of key genes. A total of 79 DEGs were identified. Enrichment analyses revealed that the DEGs are closely related to tissue invasion and metastasis of cancer. Subsequently, 14 hub genes including ESRP1, AP1M2, CLDN4, and CLDN7 were detected. Kaplan–Meier analysis indicated that the low expression of CLDN7 and GNAS was related to the worse overall survival in patients with ChRCC. Univariate Cox analysis showed that CLDN7 might be a helpful biomarker for ChRCC prognosis. Subgroup analysis revealed that the expression of CLDN7 showed a downtrend with the development of the clinical stage, topography, and distant metastasis of ChRCC. GSEA analysis identified that cell adhesion molecules cams, B cell receptor signaling pathway, T cell receptor signaling pathway, RIG-I like receptor signaling pathway, Toll-like receptor signaling pathway, and apoptosis pathway were associated with the expression of CLDN7. In conclusion, ESRP1, AP1M2, CLDN4, PRSS8, and CLDN7 were found to distinguish ChRCC from RO. Besides, the low expression of CLDN7 was closely related to ChRCC progression and could serve as an independent risk factor for the overall survival in patients with ChRCC.

## 1. Introduction

Chromophobe renal cell carcinoma (ChRCC) was the third most common histologic subtype of renal cell carcinoma, accounting for about 5%–10% of the total cases of renal cell carcinoma [1]. Compared with renal oncocytoma (RO), the second most common benign renal neoplasm, ChRCC was understood to be a malignant tumor with a high possibility for metastatic spread and death [2]. Surgical intervention was the standard treatment for RO, while no standard therapy has been identified for advanced ChRCC. However, these two types of renal tumors shared histologic, immunohistochemical, and ultrastructural features, which added difficulties in accurately distinguishing the two entities [3]. Fluorescence in situ hybridization, proteomics, and cytogenetics might be useful techniques but they were costly and not easily available. There were still clinical dilemmas in precisely differentiating ChRCC from RO. Therefore, techniques with the confident exact diagnosis of these two entities needed more investigations, especially via noninvasive means.

The gene mutation was known to play a key role in the occurrence, development, and prognosis of diverse diseases. A large number of studies have shown that gene biomarkers were widely used in various disease diagnoses and targeted treatments, like digestive system neoplasms [4], Alzheimer's diseases [5], and diabetes mellitus [6]. Moreover, a better understanding of the molecular mechanisms of tumors helped discover the more efficient strategies for the management. Microarray technology showed an increasingly powerful function on genome-wide scanning and new key genes discovery in special diseases. Jon Jones discussed the transcriptional profiling with oligonucleotide microarrays (22,283 genes) in 49 RCC tumors and explored the biomarkers associated with tumor progression and metastases [7], providing abundant resources for further investigation. However, the results of individual microarray analysis seemed to be disputable due to its false-positive rates. To identify new DEGs as effective biomarkers for the diagnosis in ChRCC and RO, we merged multichip mRNA microarray datasets which were downloaded from Gene Expression Omnibus (GEO) and used The Cancer Genome Atlas (TCGA) data to analyze the prognostic value of key genes in ChRCC. All the samples were originated from tumor tissues.

## 2. Materials and Methods

### 2.1. GEO Datasets Collection

GEO was a functional genomics data platform [8], collecting gene expression data, chips, and microarrays from various tumor samples and nontumor samples (available online: https://www.ncbi.nlm.nih.gov/geo/). In our study, three mRNA microarray datasets were eligible for data merging after screening. GSE12090 (9 ChRCC samples and 9 RO samples) [9], GSE19982 (15 ChRCC samples and 15 RO samples) [10], and GSE8271 (10 ChRCC samples and 10 RO samples) [11] were obtained from GEO. Selection criteria were as follows: (i) each dataset contained the human gene expression profiles of ChRCC and RO; (ii) ChRCC and RO tissues samples were more than 5 in each dataset, respectively; (iii) series matrix file of each dataset was available and intact.

### 2.2. Data Batch Normalization and Identification of DEGs

To remove batch effects which might originate from diverse laboratory conditions, reagent lots, and personnel differences and get a standardized gene expression matrix, we used the R (R version 3.6.0) package SVA [12] with ComBat function to normalize data. DEGs between ChRCC and RO samples were screened using the Limma package [13]. The cutoff criteria were set as follows: |log Fold Change| > 1 and adjusted *P* value <0.05. Visualized volcano plot and heat map of DEGs were implemented by R.

### 2.3. Functional Enrichment Analysis of DEGs

The Database for Annotation, Visualization, and Integrated Discovery (DAVID) [14] was a well-known online biological information database for data analysis (available online: https://david.ncifcrf.gov/). We used DAVID to execute gene ontology (GO) and Kyoto Encyclopedia of Genes and Genomes (KEGG) pathway enrichment analyses of DEGs. Adjusted *P* < 0.05 showed statistical significance. The R package GOplot [15], DOSE [16], and ClusterProfiler [17] were utilized to implement visualized figures of GO and KEGG enrichment analyses.

### 2.4. PPI Network Construction and Key Modules Screening

We utilized the Search Tool for the Retrieval of Interacting Genes (STRING) (version 10.5) [18] in the construction of the PPI network of DEGs (available online: https://string-db.org/). DEGs with a combined score ≥0.4 were eligible for constructing the relational network, which was visualized by Cytoscape (version 3.7.0) [19]. Subsequently, we used Molecular Complex Detection (MCODE) [20] (version 1.4.2) to attain key modules of PPI network and the screening conditions were set as follows: degree cutoff = 2, MCODE scores > 5, Max depth = 100, k-score = 2, and node score cutoff = 0.2.

### 2.5. Hub Genes Verification Using Oncomine Analysis

Oncomine was an accessible online tool for discovering new biomarkers in various tumor microarray databases (https://www.oncomine.org/). In the present study, Oncomine database was used for validating the different expressions of hub genes between ChRCC and RO tissues. Three available studies were selected, that is, Yusenko's study [21], Bittner's study (not published), and Jone's study [7].

### 2.6. Survival Analysis of Key Genes by TCGA

65 genes expression datasets and relative clinical information were downloaded from the TCGA website for the Kidney Chromophobe projects (TCGA-KICH) (available online: https://portal.gdc.cancer.gov/). The association between key genes and the overall survival of ChRCC patients was done by the Kaplan–Meier method. Log-rank *P* < 0.05 showed statistical significance. We analyzed the relationship between clinical features and key genes using the Wilcoxon signed-rank test and the logistic regression. Univariate Cox analysis and multivariate Cox analysis were utilized for comparing the influence of key genes expression on survival along with other clinical characteristics. All statistical analyses were conducted using R [22].

### 2.7. Gene Set Enrichment Analysis

GSEA was a computing method that identified whether an a priori defined set of genes had statistical significance and concordant differences between two biological states [23] (available online: http://software.broadinstitute.org/gsea/index.jsp). The gsea-3.0.jar version was used for analysis. Tumor tissue samples were divided into high and low expression groups according to the median expression level of CLDN7, and then the effect of the CLDN7 expression on various gene sets was analyzed by GSEA with the enrichment of MSigDB Collection (h.all.v6.2.symbols.gmt) [24]. Gene set permutations were performed 1000 times for each analysis. Absolute value of normalized enrichment score (NES) > 1, NOM *P* value <0.05, and FDR *q* value <0.05 were considered as statistical significance.

## 3. Results

### 3.1. Identification of DEGs between ChRCC and RO

**Figure 1:**
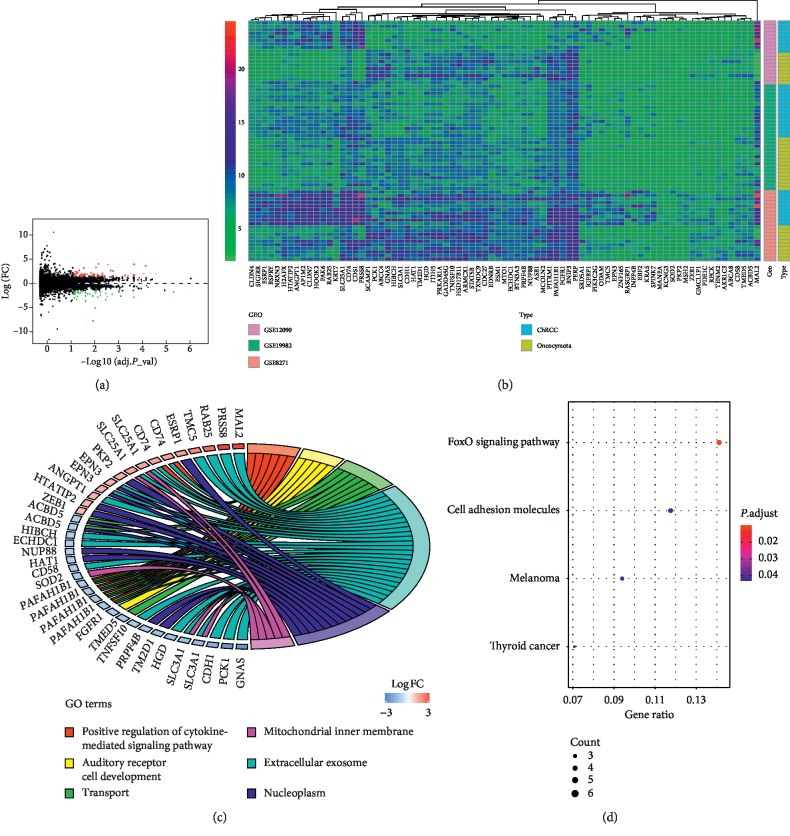
DEGs identification and function enrichment of DEGs. (a) Volcano map of DEGs between ChRCC and RO tissues. The red points represent upregulated genes and the green points represent downregulated genes. (b) Heat map of the 79 DEGs based on the |log Fold Change| > 1 and adjusted *P* value <0.05. (c) GO terms in the enrichment analysis of the 79 DEGs. (d) The KEGG pathways in the enrichment analysis of the 79 DEGs.

After batch normalization and analysis of the selected datasets (GSE12090, GSE19982, and GSE8271), 79 DEGs were identified in ChRCC, including 33 significant upregulated genes and 46 downregulated genes, compared to RO (Figures 1(a) and 1(b), and [Supplementary-material supplementary-material-1]).

### 3.2. GO and KEGG Enrichment Analyses of DEGs

Enrichment analysis was carried out using online DAVID and the results were visualized by R language. GO analysis showed that changes in the biological process (BP) of 79 DEGs were significantly enriched in positive regulation of cytokine-mediated signaling pathway, auditory receptor cell development, and transport. Changes in cell component (CC) were prominently enriched in extracellular exosome, nucleoplasm, and mitochondrial inner membrane (Figure 1(c) and [Supplementary-material supplementary-material-1]). The analysis of Molecular Function (MF) enrichment showed no statistical significance. KEGG enrichment results reported that 79 DEGs were mainly enriched in FoxO signaling pathway, cell adhesion molecules, melanoma, and thyroid cancer (Figure 1(d) and [Supplementary-material supplementary-material-1]).

### 3.3. PPI Network Construction and Key Modules Screening

**Figure 2:**
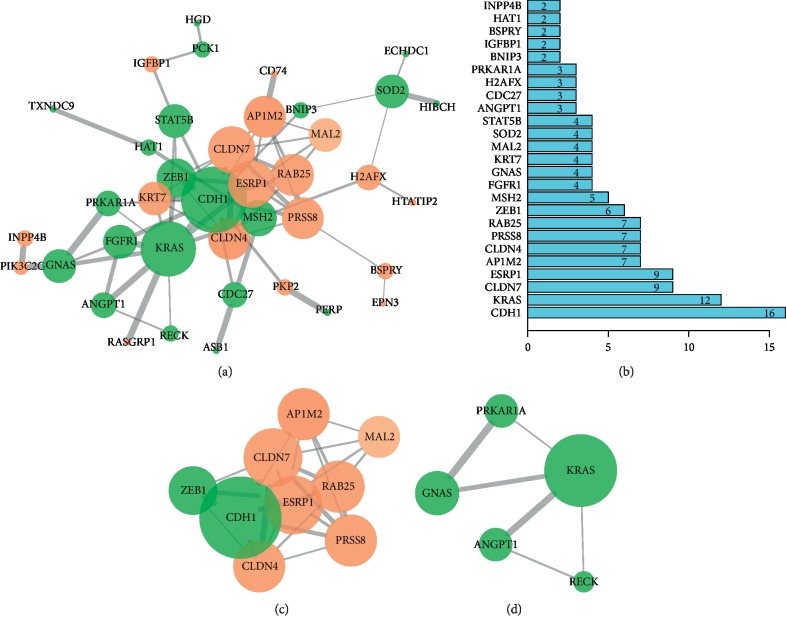
PPI network of 79 DEGs and the key modules selection. (a) PPI network of DEGs according to the interaction score ≥0.4. (b) The histogram of the top 25 genes with the number of edges. (c) Module 1 of the PPI network is selected using MCODE software in Cytoscape. (d) Module 2 of the PPI network. Upregulated genes are marked in light red and downregulated genes are marked in light green. Note: the thicker the line, the higher the combined score; the more the edge, the bigger the circle.

The result of PPI network of DEGs was illustrated in Figure 2(a). A total of 43 nodes with 77 edges were reflected in this established network system. The statistical results in Figure 2(b) indicated that CDH1, KRAS, CLDN7, and ESRP1 were the most important genes in the network. After screening the modules of the network by Cytoscape software, two significant modules were eligible. Module 1 contained 9 hub genes (CLDN7, ESRP1, ZEB1, CLDN4, CDH1, PRSS8, RAB25, MAL2, and AP1M2) (Figure 2(c)) as well as 5 hub genes (GNAS, ANGPT1, RECK, KRAS, and PRKAR1A) in module 2 (Figure 2(d)).

### 3.4. Validation of the Hub Genes

**Figure 3:**
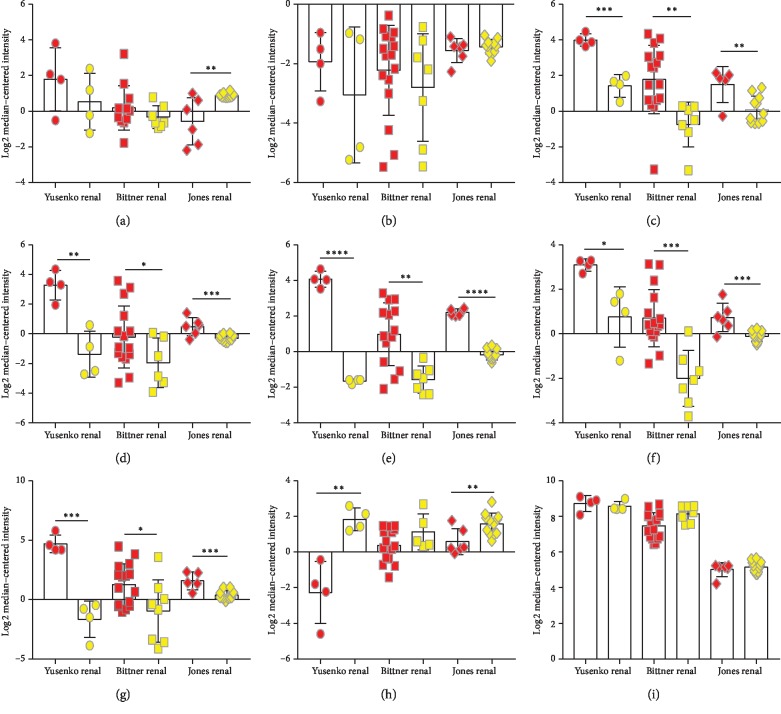
The results of hub genes expression in ChRCC and RO tissues from Oncomine data. Each plot denotes the log2 median-centered intensity of the gene expression of every single sample. The *t*-test was performed on the relevant results (^*∗*^*P* < 0.05, ^*∗∗*^*P* < 0.01, ^*∗∗∗*^*P* < 0.001, and ^*∗∗∗∗*^*P* < 0.0001). (a) CDH1 expression. (b) KRAS expression. (c) CLDN7 expression. (d) ESRP1 expression. (e) AP1M2 expression. (f) CLDN4 expression. (g) PRSS8 expression. (h) ZEB1 expression. (i) GNAS expression.

After the validation using Oncomine online data, we found that CLDN7, ESRP1, AP1M2, CLDN4, PRSS8, and ZEB1 were differentially expressed between ChRCC and RO (*P* < 0.05), which were consistent with the results performed by GEO data (Figure 3).

### 3.5. Survival Analysis of Hub Genes

**Figure 4:**
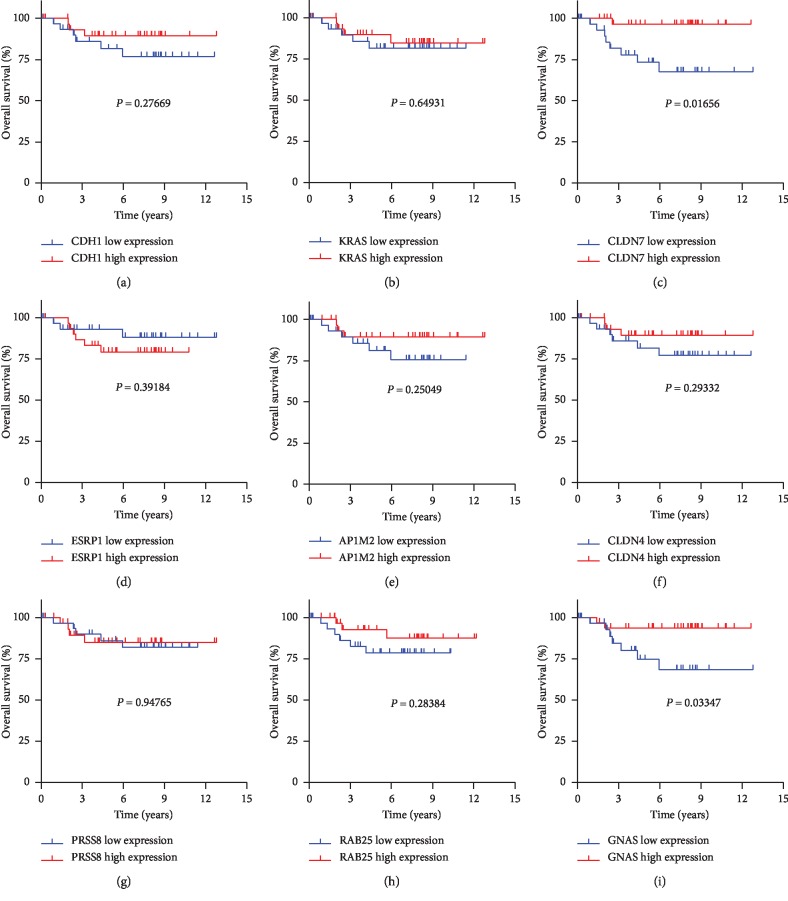
Overall survival in patients with ChRCC. The Kaplan–Meier curve is performed according to the high and low expressions of each huge gene in ChRCC. *P* < 0.05 shows statistical significance.

Clinical data (65 ChRCC samples) were downloaded from TCGA. To analyze the overall survival in patients with ChRCC, the Kaplan–Meier curve was performed according to the high and low expressions of each hub gene. The results suggested that patients with low CLDN7 (*P*=0.017) or GNAS (*P*=0.033) expression had significantly worse overall survival than those with high expression (Figure 4).

### 3.6. The Prognostic Value of Significant Hub Genes in Patients with ChRCC

**Figure 5:**
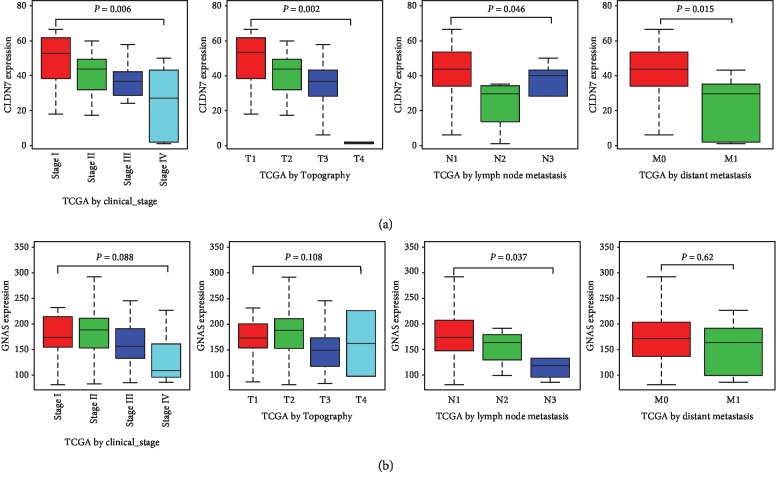
Relationship between gene expression and clinicopathologic characteristics. (a) Association between CLDN7 expression and clinical stage, topography, lymph nodes, and distant metastasis. (b) Association between GNAS expression and clinical stage, topography, lymph nodes, and distant metastasis. *T*: topography; *N*: lymph node; *M*: distant metastasis.

The two aforementioned hub genes (CLDN7 and GNAS) associated with the overall survival were selected for further prognostic evaluation in patients with ChRCC. Subgroup analyses suggested that CLDN7 showed a decreasing trend with the development of the clinical stage, topography, and distant metastasis (Figure 5(a)). The downtrend of GNAS was associated with the progress of lymph nodes metastasis but not clinical stage, topography, and distant metastasis (Figure 5(b)). Univariate analysis indicated that tumor topography, lymph node metastasis, and distant metastasis as well as the low expression of CLDN7 (HR = 0.97, 95% CI (0.932–0.990)) (Table 1) were independent risk factors for the overall survival in the patients with ChRCC. However, multivariate analysis adjusted by age, gender, clinical stage, topography, lymph node, and distant metastasis indicated that CLDN7 no longer achieved statistical significance, but distant metastasis and lymph nodes metastasis remained statistical significance.

### 3.7. CLDN7-Related Signaling Pathway Identification Using GSEA

**Figure 6:**
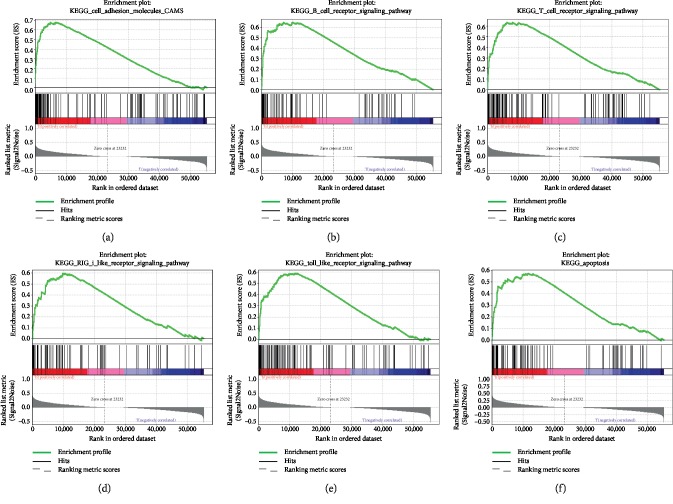
Enrichment plots by GSEA. Genes related to cell adhesion molecules cams, B cell receptor signaling pathway, T cell receptor signaling pathway, RIG-I like receptor signaling pathway, Toll-like receptor signaling pathway, and apoptosis pathway are differentially enriched in ChRCC cases with high CLDN7 expression.

GSRA was applied to analyze signaling pathways activated in ChRCC according to the CLDN7 expression. The results showed that cell adhesion molecules cams, B cell receptor signaling pathway, T cell receptor signaling pathway, RIG-I like receptor signaling pathway, Toll-like receptor signaling pathway, and apoptosis pathway were associated with the expression of CLDN7 (Figure 6). The details were reported in Table 2.

NES: normalized enrichment score; NOM: nominal; FDR: false discovery rate. Gene sets with NOM *P* value <0.05 and FDR *q* value <0.05 are considered as statistical significance.

## 4. Discussion

Conventional methods are sometimes hard to distinguish ChRCC from RO due to the overlap of morphological and ultrastructural features. ChRCC is a malignant tumor with higher mortality than RO, so next-generation diagnostic methods with high efficiency and high accuracy are urgently demanded. It has been certified that gene mutations had significant effects on the occurrence, development, and prognosis of tumors. The expression of distinctive genes in some diseases not only benefits the early diagnosis but also provides targeted therapy. Therefore, it is of great value to explore new diagnostic methods from the genetic perspective. A previous report [25] showed that the deletion of ERBB4 and RB1 might provide a sensitive and specific method to differentiate ChRCC from RO. The study by Ehsani et al. [26] showed that BCA2 could be a biomarker that might be used in the distinction between RO and its mimickers. However, none of these was entirely specific. Recently, microarray technology has shown a powerful potential in exploring the genetic alteration in different tumor tissues and it has been widely utilized in identifying new biomarkers in colorectal cancer [27], breast cancer [28], and gastric carcinoma [29].

To reduce false-positive rates which might originate from diverse laboratory conditions, reagent lots, and other uncontrolled conditions, 3 mRNA microarray datasets were merged to gain DEGs between ChRCC tissues and RO tissues. Compared with RO, there were 79 DEGs identified in ChRCC, including 33 upregulated genes and 46 downregulated genes. KEGG and GO enrichment analyses were implemented to explore the interacted function of the DEGs. The results of GO analysis showed that the DEGs (CD74, PAFAH1B1) were associated with the biological process of the cytokine-mediated signaling pathway, suggesting that these two genes may have the potential to stimulate tumor growth and progression [30]. Moreover, cell component analysis noted that the vast majority of DEGs, like MAL2, PRSS8, RAB25, GNAS, and CDH1, were mainly located in the extracellular exosome. Previous reports have certified that exosomes can act as functional mediators in cell interaction, resulting in cancer metastasis [31]. Four significant pathways were found after KEGG enrichment analyses. FoxO signaling pathway [32], cell adhesion molecules pathway [33], and Rap1 signaling pathway [34] have been proved to be closely related to tissue invasion and metastasis of cancer.

Two key modules including 14 hub genes were selected after screening by STRING and MCODE. CDH1, KRAS, and CLDN7 seemed to locate at the hub of the network because the numbers of edges linked to these genes were the largest. Cadherin-1, calcium-dependent cell adhesion proteins, was the translation product of CDH1. The function of cadherin-1 was to promote adhesion between adjacent cells and played a key role in cell development, tissue maintenance, and tumor inhibition [35]. Our study found that the expression of CDH1 was lower in ChRCC compared with RO, suggesting that CDH1 inhibition might be one of the key factors for early metastasis in ChRCC. Moreover, the study of Costa et al. reported that CDH1 methylation levels varied from different renal cell tumors and the results pointed out that CDH1 hypermethylation levels were significantly lower in ChRCC compared with RO [36]. GTPase KRas, the protein encoded by KRAS, showed a powerful function in the regulation of cell proliferation. Besides, KRAS mutation in colorectal cancer has been reported and KRAS/BRAF genes mutation might make EGFR inhibitors ineffective [37]. Results of Kozma et al.'s investigation showed that KRAS amplification was associated with tumor size and the pathological grade, indicating that KRAS amplification might account for a more rapid progression of renal clear cell cancer [38]. CLDN7 (Claudin 7), a member of the claudin family, played an important role in the tight junction formation and function of the intercellular space [39]. Claudin family proteins have been declared to be expressed differently in diverse tumor tissues and CLDN7 was particularly relevant to gastric cancer [40], colon cancer [41], and pancreatic cancer [42]. In our study, the expression of CLDN7 in ChRCC was three times higher than the expression in RO, indicating that CLDN7 had the potential to differentiate ChRCC from RO. The result was further supported by a meta-analysis including three observational studies [43]. Overall, the majority of key genes found by pooled microarray datasets in our study were somewhat similar compared with previous reports.

To provide more significant clinical values, we further performed the prognostic analyses of each hub gene. Clinical information and gene expression matrix were downloaded from TCGA database. Results showed that the overall survival was correlated with CLDN7 and GNAS but not with the other hub genes (CDH1, KRAS, ESRP1, AP1M2, CLDN4, PRSS8, and RAB25). Although Li et al.'s study reported that the downregulated expression of CLDN7 was correlated with the progression and poor prognosis in CCRCC [44], the relationships between CLDN7, GNAS, and the prognosis of ChRCC were firstly reported in our study. Even more remarkably, subgroup analyses revealed that CLDN7 showed a decreasing trend with the progress of the clinical stage, topography, and distant metastasis in ChRCC. All these factors were convinced to be the worse prognosis in cancer. In addition, Univariate Cox analyses indicated that the expression of CLDN7 might be a significant biomarker for ChRCC prognosis but not GNAS. However, the result did not achieve statistical significance after multivariate Cox analyses. More investigations were required to certify the function of CLDN7 in ChRCC prognosis.

The association between CLDN7 and cancer prognosis varied from diverse cancers. The low expression of CLDN7 was found to be correlated with breast cancer grade and metastasis [45] as well as colon cancer progression [46]. Some studies showed inverse results, indicating that the overexpression of CLDN7 increased proliferation and migration in gastric adenocarcinoma [47] and promoted invasion in ovarian cancer [48]. However, the mechanism of CLDN7 in cancer progression and metastasis remained unknown. The downregulation of CLDN7 might decrease the expression of E-cadherin, leading to the loss of epithelial architecture, increasing invasion [49]. Besides, Li et al.'s study reported that CLDN7 might suppress cell growth and metastasis by inducing cell apoptosis and inhibiting the epithelial-mesenchymal transition pathway in CCRCC [44]. To further explore the mechanism of CLDN7 in ChRCC, we performed GSEA. The results showed that cell adhesion molecules cams, B cell receptor signaling pathway, T cell receptor signaling pathway, RIG-I like receptor signaling pathway, Toll-like receptor signaling pathway, and apoptosis pathway were differentially enriched in high CLDN7 expression phenotype, offering a potential mechanism for further investigations.

Overall, bioinformatic analysis using mRNA microarray datasets from GEO and TCGA indicated that CLDN7 might provide evidence for the diagnostic and prognostic value in ChRCC. However, our study was performed at a bioinformatics level and the results were limited to the number of microarray datasets. Clinical investigation and biological experiments were imperative.

## 5. Conclusion

14 hub genes, especially ESRP1, AP1M2, CLDN4, and CLDN7, were found to differentiate ChRCC from RO. Besides, the low expressions of CLDN7 are related to tumor progression and high overall survival rates in patients with ChRCC. CLDN7 can serve as a helpful biomarker in the diagnostic and prognostic evaluations of ChRCC.

## Figures and Tables

**Table 1:** Univariate analysis and multivariate analysis of the correlation of CLDN7 expression with overall survival among ChRCC patients.

Parameter	Univariate analysis			Multivariate analysis		
	HR	95%CI	*P*	HR	95%CI	*P*
Age	1.06	0.999–1.117	0.055			
Gender	1.54	0.385–6.189	0.540			
Stage	7.63	2.616–22.23	0.000^*∗*^	1.282	0.171–9.61	0.0509
*T*	10.11	2.155–47.42	0.003^*∗*^	1.302	0.104–16.33	0.0838
*M*	23.67	4.649–120.543	0.000^*∗*^	4.628	1.484–44.27	0.024^*∗*^
*N*	7.44	3.138–17.659	0.000^*∗*^	8.744	1.474–51.88	0.017^*∗*^
CLDN7	0.97	0.932–0.990	0.017^*∗*^	0.985	0.921–1.05	0.668

*T*, Topography; *N*, lymph node; *M*, distant metastasis; HR, hazard ratio; CI, confidence interval. ^*∗*^*P* < 0.05 shows statistical significance.

**Table 2:** Relative pathways associated with the expression of CLDN7.

Name	ES	NES	NOM *P* value	FDR *q* value
KEGG_CELL_ADHESION_MOLECULES_CAMS	0.68	1.9	0.000	0.033
KEGG_B_CELL_RECEPTOR_SIGNALING_PATHWAY	0.65	1.83	0.002	0.031
KEGG_T_CELL_RECEPTOR_SIGNALING_PATHWAY	0.63	1.82	0.000	0.029
KEGG_RIG_I_LIKE_RECEPTOR_SIGNALING_PATHWAY	0.60	1.79	0.004	0.025
KEGG_TOLL_LIKE_RECEPTOR_SIGNALING_PATHWAY	0.59	1.72	0.004	0.041
KEGG_APOPTOSIS	0.57	1.70	0.006	0.051

NES: normalized enrichment score; NOM: nominal; FDR: false discovery rate. Gene sets with NOM *P* value <0.05 and FDR *q* value <0.05 are considered as statistical significance.

## Data Availability

The data used to support our results are available at the GEO (https://www.ncbi.nlm.nih.gov/geo/), TCGA (https://portal.gdc.cancer.gov/), and Oncomine (https://www.oncomine.org/).

## Supplementary Materials

Supplementary Materials Table S1: differentially expressed genes in the three expression profiles. Table S2: GO and KEGG analyses of differentially expressed genes in ChRCC.
