# Supplementary material for: Identification of Key Genes and Prognostic Analysis between Chromophobe Renal Cell Carcinoma and Renal Oncocytoma by Bioinformatic Analysis
Source: Biomed Res Int. 2020 Jan 9;2020:4030915. doi: 10.1155/2020/4030915 (PMC6977339; doi:10.1155/2020/4030915)
Supplement: Supplementary Materials — Table S1: differentially expressed genes in the three expression profiles. Table S2: GO and KEGG analyses of differentially expressed genes in ChRCC. [file 4030915.f1.pdf]

Table S1. DEGs of the three expression profiles.

DEGs	Genes Name
Up-regulated	SIGIRR,CLDN4,TMC5,ZNF165,ZEB1,EPN3,ESRP1,CLDN7,HOOK2,KRT7,BSPRY,PKP2,AP1M2,SPINK7,PRSS8,HTATIP2,MAL2,KRAS,CD74,RAB25,ODAM,NRXN3,PIK3C2G,SLC25A1,IGFBP1,EBF2,INPP4B,H2AFX,CDS1,ANGPT1,SRD5A1,PAK6,RASGRP1
Down-regulated	KCNG3,MANEA,MCOLN2,TENM2,SOD2,ABCA8,GMCL1P1,BTN3A3,MSH2,RECK,HSD17B11,ECHDC1,CDH1,PCK1,MTO1,SLC3A1,PDE1C,ACBD5,GNAS,TMED5,ASB1,CD58,AKR1C3,NUP88,TXNDC9,PRKAR1A,ITIH5,HIBCH,STAT5B,CDC27,PAFAH1B1,PRPF4B,ABCC4,ESM1,TNFSF10,TM2D1,FGFR1,HAT1,SCAMP1,EDNRB,PERP,BNIP3,GADD45G,ARMCX1,PITRM1,HGD

Table S2. The GO and KEGG analyses of DEGs in ChRCC.

GO Term	Description	Count	Genes	P-value
GO:0001961	Positive regulation of cytokine-mediated signaling pathway	2	PAFAH1B1, CD74	0.0241
GO:0060117	Auditory receptor cell development	2	FGFR1, PAFAH1B1	0.0288
GO:0006810	Transport	3	TMED5, PAFAH1B1, ACBD5	0.0399
GO:0070062	Extracellular exosome	18	EPN3,MAL2,TMC5,ECHDC1, HGD,CDH1,SLC3A1,CD74,PCK1,PRSS8,TNFSF10,CD58,SLC25A1,RAB25,GNAS, PAFAH1B1, ANGPT1,HIBCH	0.010
GO:0005654	Nucleoplasm	12	SCAMP1,EPN3,HTATIP2,PRPF4B,TM2D1,NUP88,PKP2,ESRP1,HAT1, ZEB1,CDC27,ACBD5	0.025
GO:0005743	Mitochondrial inner membrane	4	ABCA8,SLC25A1,SLC3A1, SOD2	0.0347
KEGG				P.adjust
hsa04068	FoxO signaling pathway	6	TNFSF10,KRAS,GADD45G,BNIP3,SOD2,PCK1	0.0107
hsa05218	Cell adhesion molecules	5	PAK6,FGFR1,KRAS,RASGRP1,ANGPT1	0.0424
hsa05216	Rap1 signaling pathway	4	FGFR1, KRAS, GNAS, CDH1	0.0424
hsa04514	Melanoma	3	FGFR1, KRAS, CDH1	0.0423
